# Data on the synthesis processes optimization of novel β-NiS film modified CdS nanoflowers heterostructure nanocomposite for photocatalytic hydrogen evolution

**DOI:** 10.1016/j.dib.2017.12.016

**Published:** 2017-12-16

**Authors:** Yu Zhang, Zhijian Peng, Shundong Guan, Xiuli Fu

**Affiliations:** a School of Engineering and Technology, China University of Geosciences, Beijing 100083, PR China; b School of Science, China University of Geosciences, Beijing 100083, PR China; c State Key Laboratory of Information Photonics and Optical Communications, and School of Science, Beijing University of Posts and Telecommunications, Beijing 100876, PR China

**Keywords:** NiS/CdS nanoflowers heterostructure, Photocatalysts, Water splitting, Hydrothermal synthesis, Optimization

## Abstract

The data presented in this article are related to a research article entitled ‘Novel β-NiS film modified CdS nanoflowers heterostructure nanocomposite: extraordinarily highly efficient photocatalysts for hydrogen evolution’ (Zhang et al., 2018) [1]. In this article, we report original data on the synthesis processes optimization of the proposed nanocomposite on the basis of their optimum photocatalytic performance together with the comparison on the results of literatures and comparative experiments. The composition, microstructure, morphology, photocatalytic hydrogen evolution and photocatalytic stability of the corresponding samples are included in this report. The data are presented in this format in order to facilitate comparison with data from other researchers in the field and understanding the mechanism of similar catalysts.

**Specifications Table**

**Table:** 

Subject area	*Chemical engineering, Environmental engineering, Materials chemistry, Materials physics*
More specific subject area	*New energy, Photocatalytic hydrogen evolution*
Type of data	*Tables, Figures*
How data was acquired	*X-ray diffraction (XRD, Rigaku D/max-RB, Japan), Field emission scanning electron microscope (FE-SEM, S4800, Japan), Transmission electron microscope (TEM, FEI Tecnai G2 F30 U-TWIN, America), Photocatalytic water splitting reaction system (Pyrex, Perfectlight, Beijing, China).*
Data format	*Raw and analyzed data*
Experimental factors	*The amounts of the used reaction resources: deionized water (constantly 50 ml), Cd(NO*_*3*_*)*_*2*_*·4H*_*2*_*O (1 mmol), CH*_*4*_*N*_*2*_*S (3 mmol), Ni(CH*_*3*_*COO)*_*2*_*·4H*_*2*_*O (0–1.2 mmol) and NaH*_*2*_*PO*_*2*_*·H*_*2*_*O (0–1.2 mmol).*
	Temperature: 180 °C for synthesizing the photocatalysts
	Reaction time: 4 h for synthesizing the photocatalysts
Experimental features	*The designed experiments included the optimization of synthesis processes and comparison on the photocatalytic hydrogen evolution*
Data source location	*The nanocomposite was grown in Beijing, China*
Data accessibility	*The data are available with this article*

**Value of the data**

• The data on the synthesis processes optimization of the β-NiS film modified CdS nanoflowers heterostructure nanocomposite (NiS/CdS NFs HSNC) could give an insight into the formation and photocatalysis mechanisms of the present composite to other researchers interested in the synthesis and application of photocatalysts.
• The data on the photocatalytic stability of the present nanocomposite could give an insight into the photocorrosion resistance of CdS NFs to other researchers interested in the engineering of photocatalysts.
• The data set can be used by researchers interested in developing new composite photocatalysts and understanding the mechanism of co-catalysts.
• The data can be used for comparison with other studies on photocatalysts.

## 1 Data

The data presented in this paper is related to a research article entitled ‘Novel β-NiS film modified CdS nanoflowers heterostructure nanocomposite: extraordinarily highly efficient photocatalysts for hydrogen evolution’ [1].

**Fig. 1:**
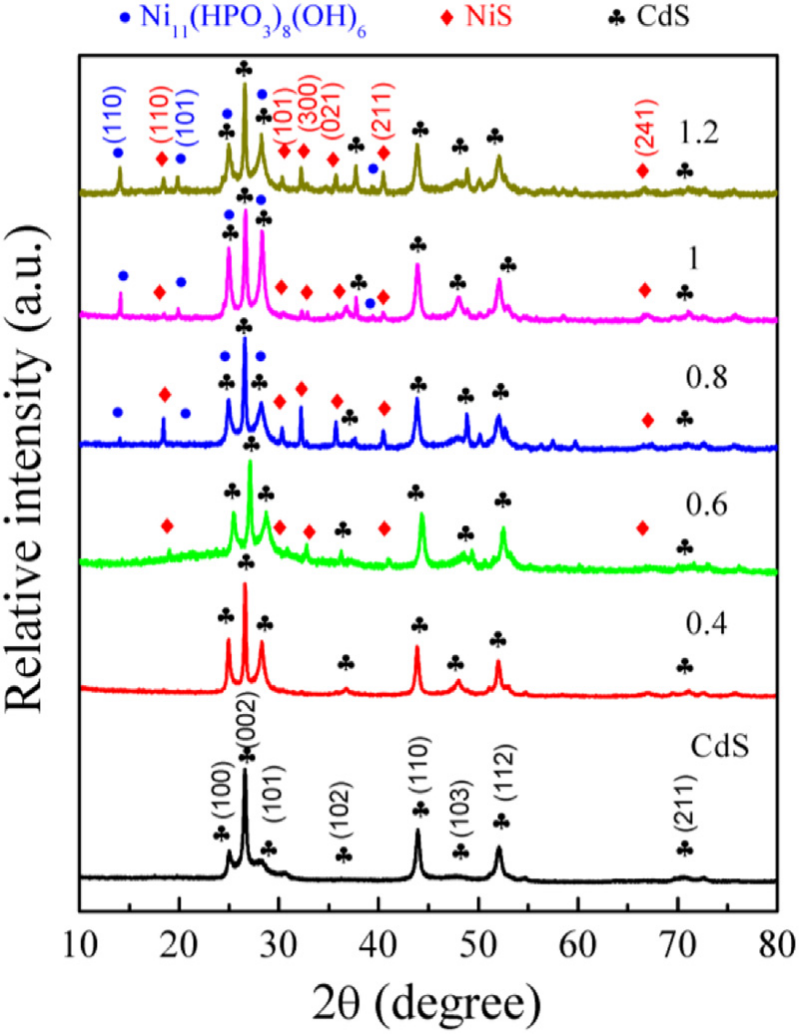
XRD patterns of pure CdS NFs, and NiS/CdS NFs HSNC prepared with 0.6 mmol of NaH_2_PO_2_·H_2_O but different FMRs of Ni/Cd: 0.4, 0.6, 0.8, 1 and 1.2, while the amount of Cd(NO_3_)_2_·4H_2_O was fixed at 1 mmol. The obtained pure CdS NFs were synthesized under the same condition as that for NiS/CdS NFs HSNCs but without Ni(CH_3_COO)_2_·4H_2_O and NaH_2_PO_2_·H_2_O (also see Table 1).

**Fig. 2:**
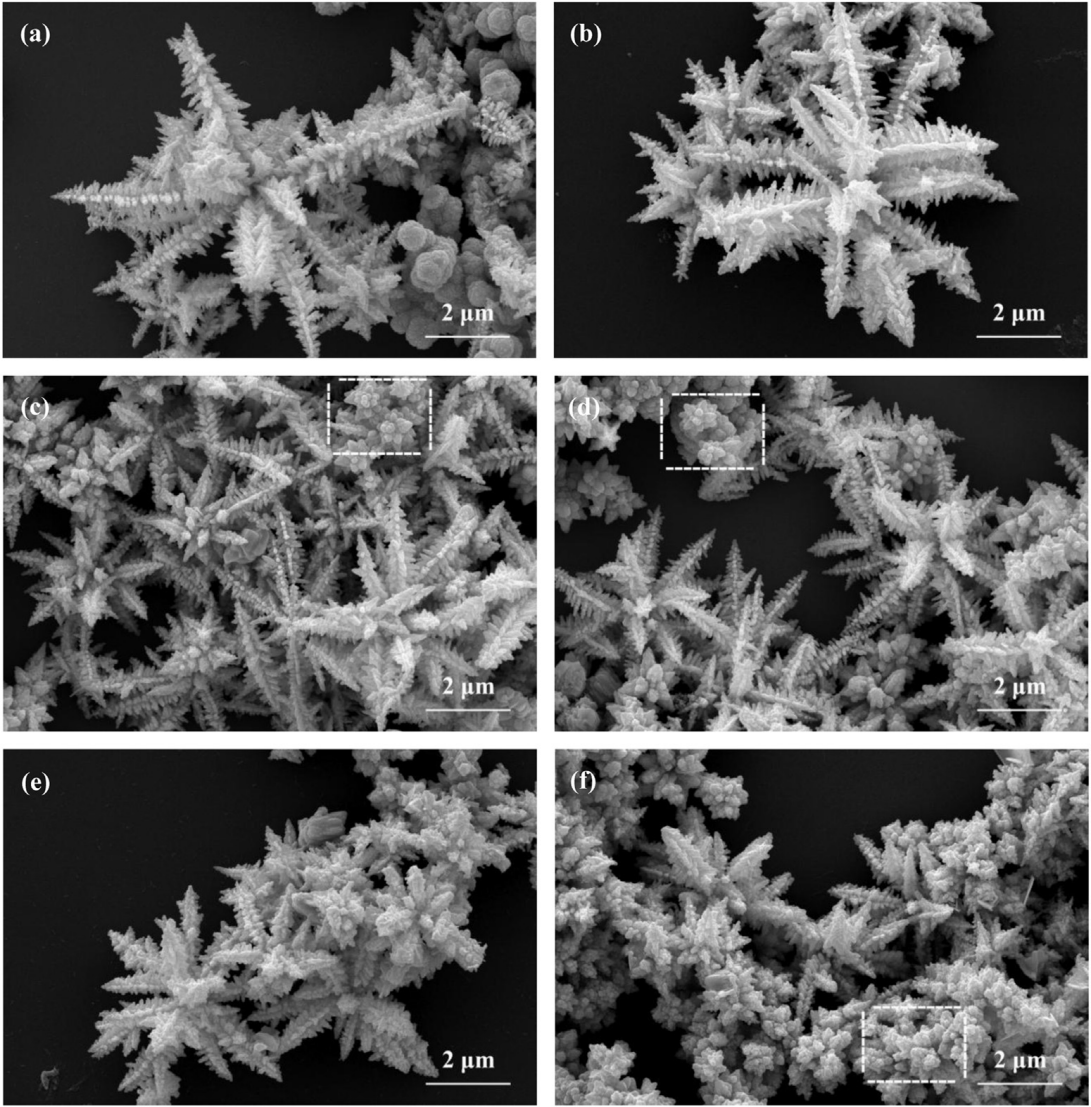
SEM images of pure CdS NFs (a) and NiS/CdS NFs HSNC prepared with 0.6 mmol NaH_2_PO_2_·H_2_O and different FMRs of Ni/Cd: (b) 0.4, (c) 0.6, (d) 0.8, (e) 1 and (f) 1.2, while the amount of Cd(NO_3_)_2_·4H_2_O was fixed at 1 mmol. The obtained pure CdS NFs were synthesized under the same condition as that for NiS/CdS NFs HSNCs but without Ni(CH_3_COO)_2_·4H_2_O and NaH_2_PO_2_·H_2_O (also see Table 1).

**Fig. 3:**
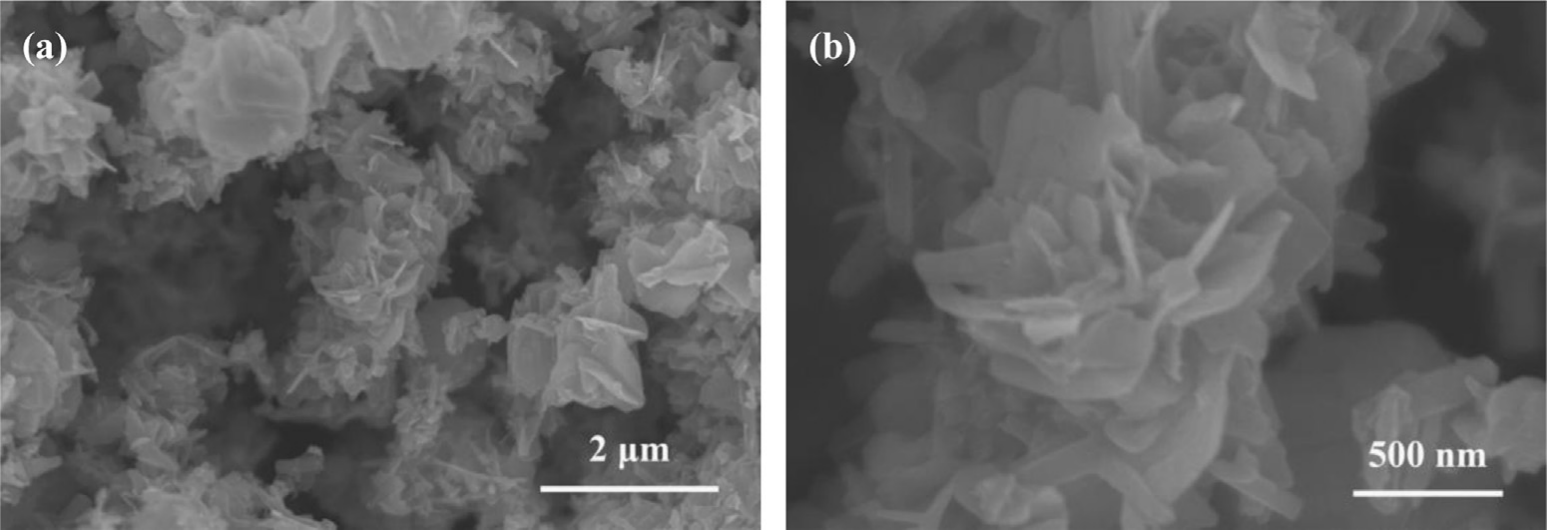
Low-magnification (a) and high-magnification (b) SEM images of pure NiS nanopowder (NP) prepared under the optimized conditions (also see Table 1e). This figure reveals that the prepared β-NiS NP is actually composed of a lot of irregular nanoplates aggregating together.

**Fig. 4:**
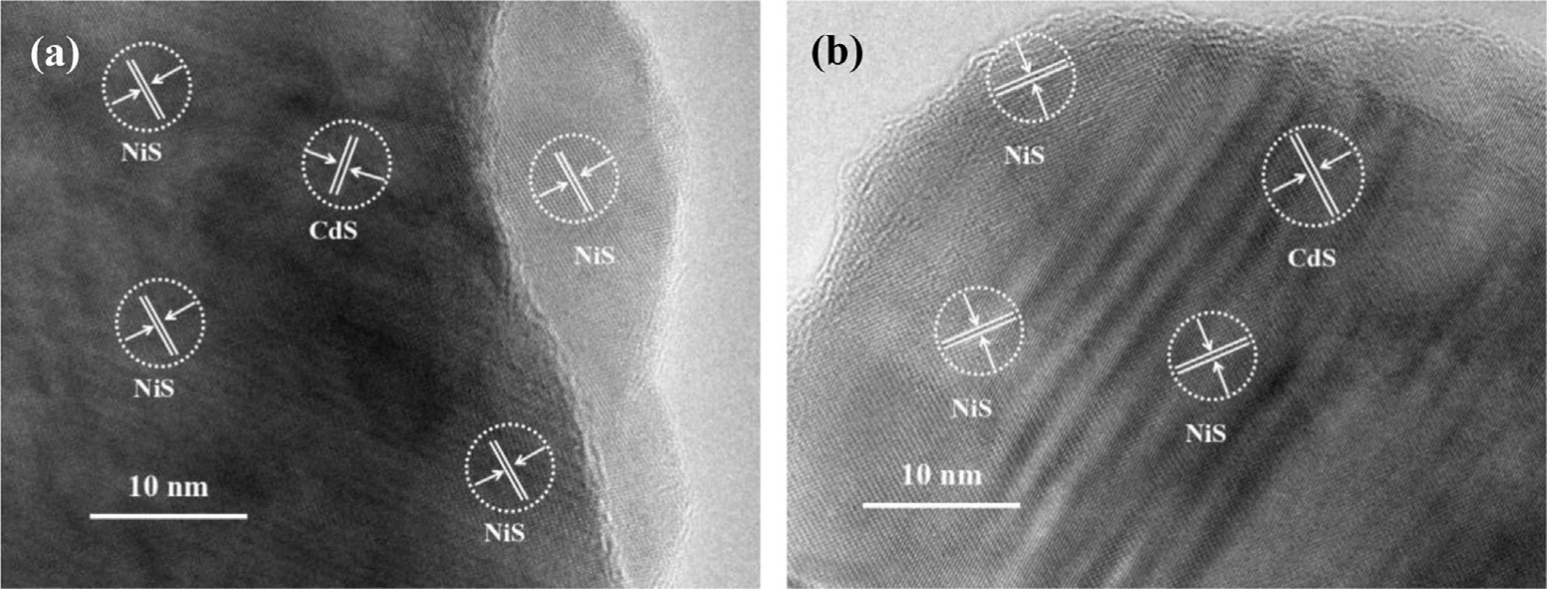
HRTEM images of the optimal NiS/CdS NFs HSNC. These HRTEM images reveal that the NiS crystals have an intimate contact with the CdS crystals, coating on the surface of CdS, because there is an atomic level bonding between them.

**Fig. 5:**
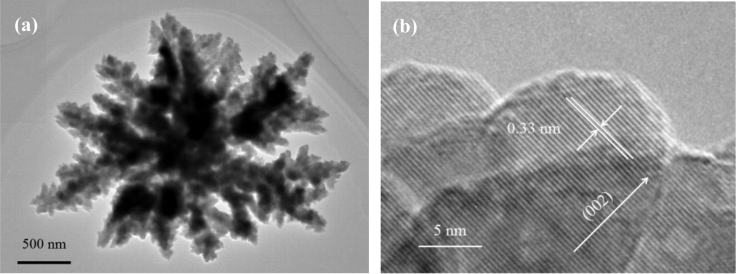
(a) TEM and (b) HRTEM images of the obtained pure CdS NFs. A flower with a diameter of about 3 μm can be observed from the TEM image of the sample (Fig. 5a). And the HRTEM image of the CdS NFs as presented in Fig. 5b reveals that the lattice spacing of 0.33 nm could be attributed to the (002) plane of hexagonal CdS, indicating that the CdS NFs have a uniform lattice fringe.

**Fig. 6:**
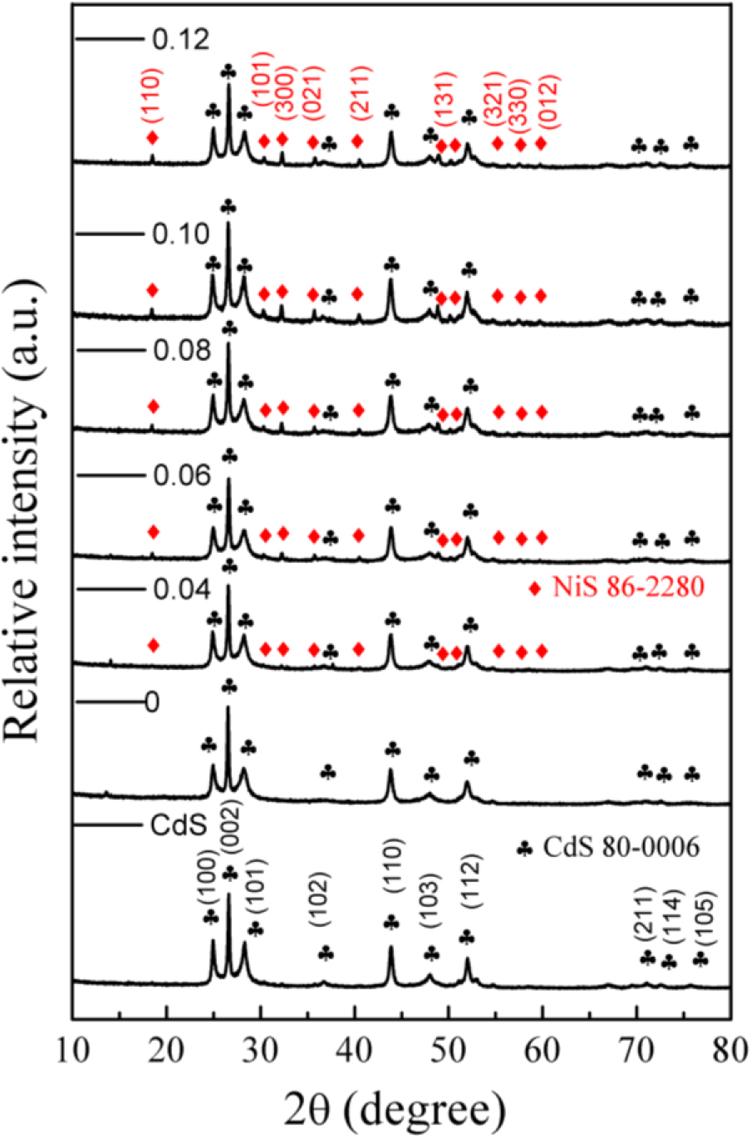
XRD patterns of pure CdS NFs and NiS/CdS NFs HSNC photocatalysts prepared with a constant Ni/Cd FMR of 0.6 and different amounts of NaH_2_PO_2_·H_2_O: 0, 0.4, 0.6, 0.8, 1.0 and 1.2 mmol, while the amount of Cd(NO_3_)_2_·4H_2_O was fixed at 1 mmol (also see Table 1).

**Fig. 7:**
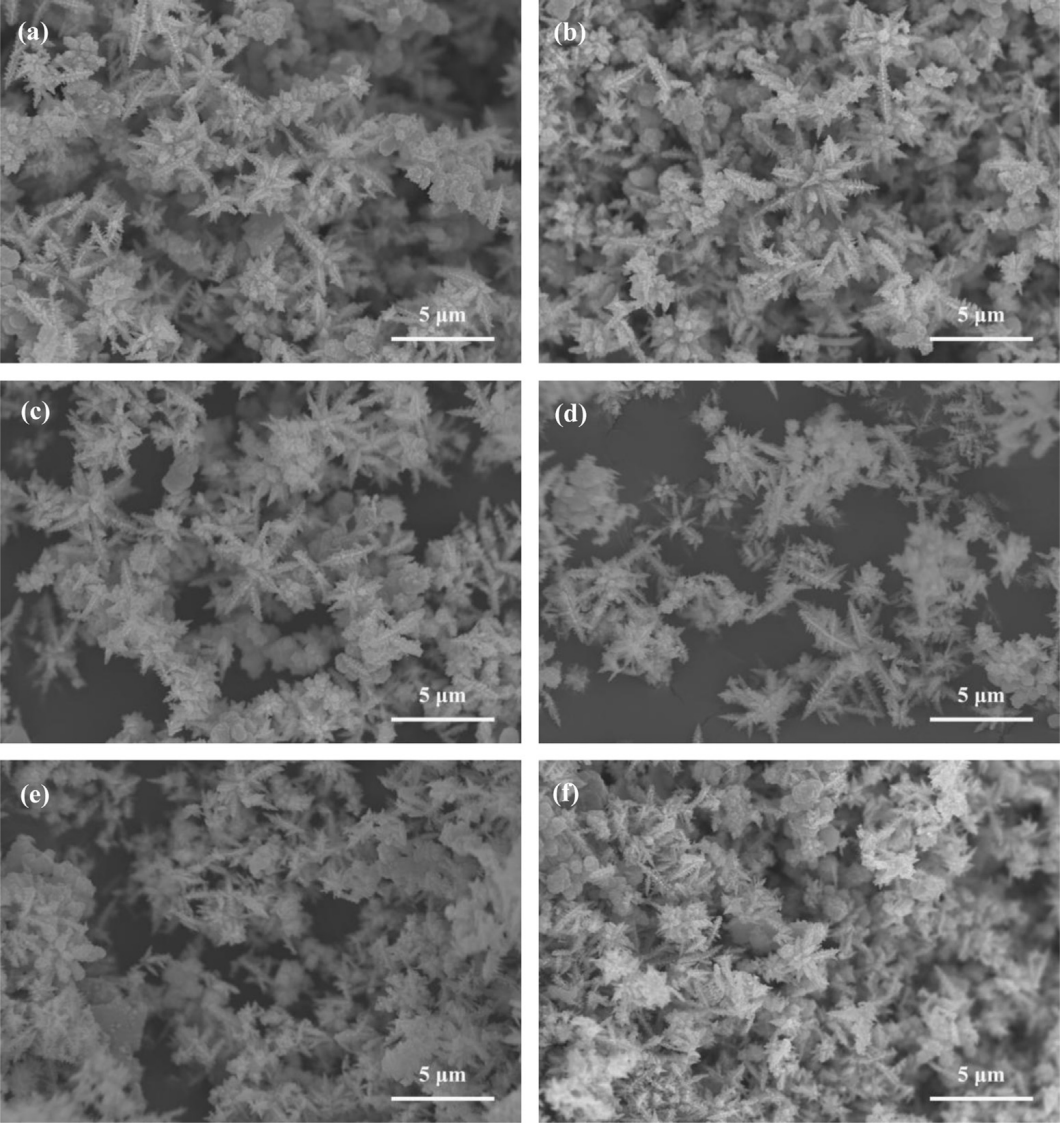
SEM images of NiS/CdS NFs HSNCs prepared with a constant Ni/Cd FMR of 0.6 and different amounts of NaH_2_PO_2_·H_2_O: (a) 0, (b) 0.4, (c) 0.6, (d) 0.8, (e) 1.0 and (f) 1.2 mmol, while the amount of Cd(NO_3_)_2_·4H_2_O was fixed at 1 mmol (also see Table 1).

**Fig. 8:**
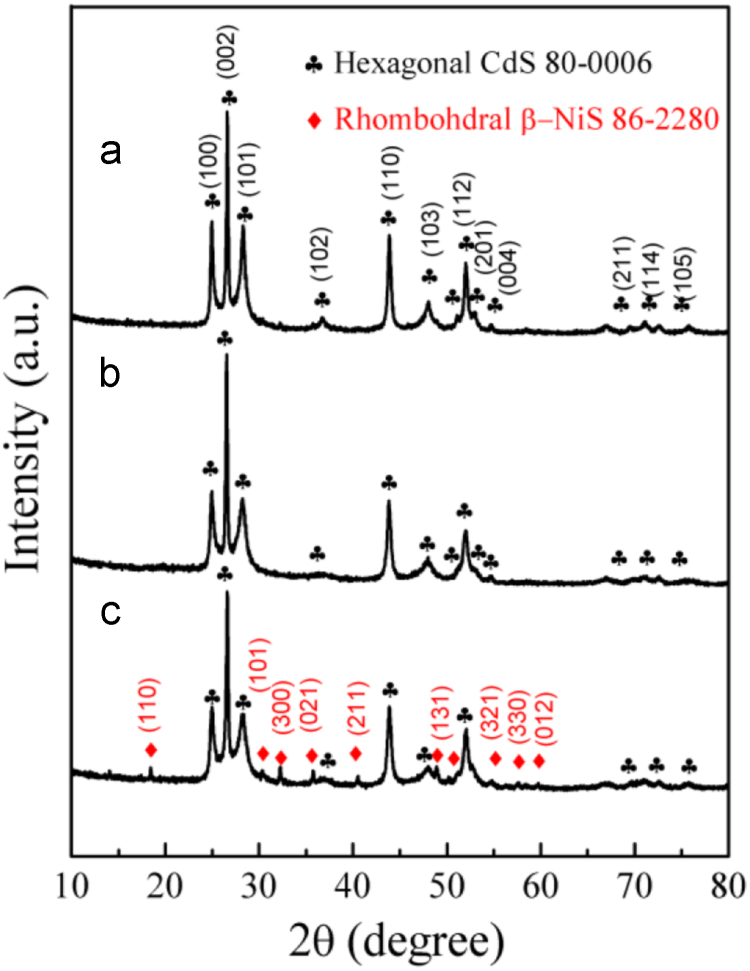
Comparison on the XRD patterns of the samples prepared under the conditions listed in Table 1a (a), Table 1b (b) and Table 1g (c).

**Fig. 9:**
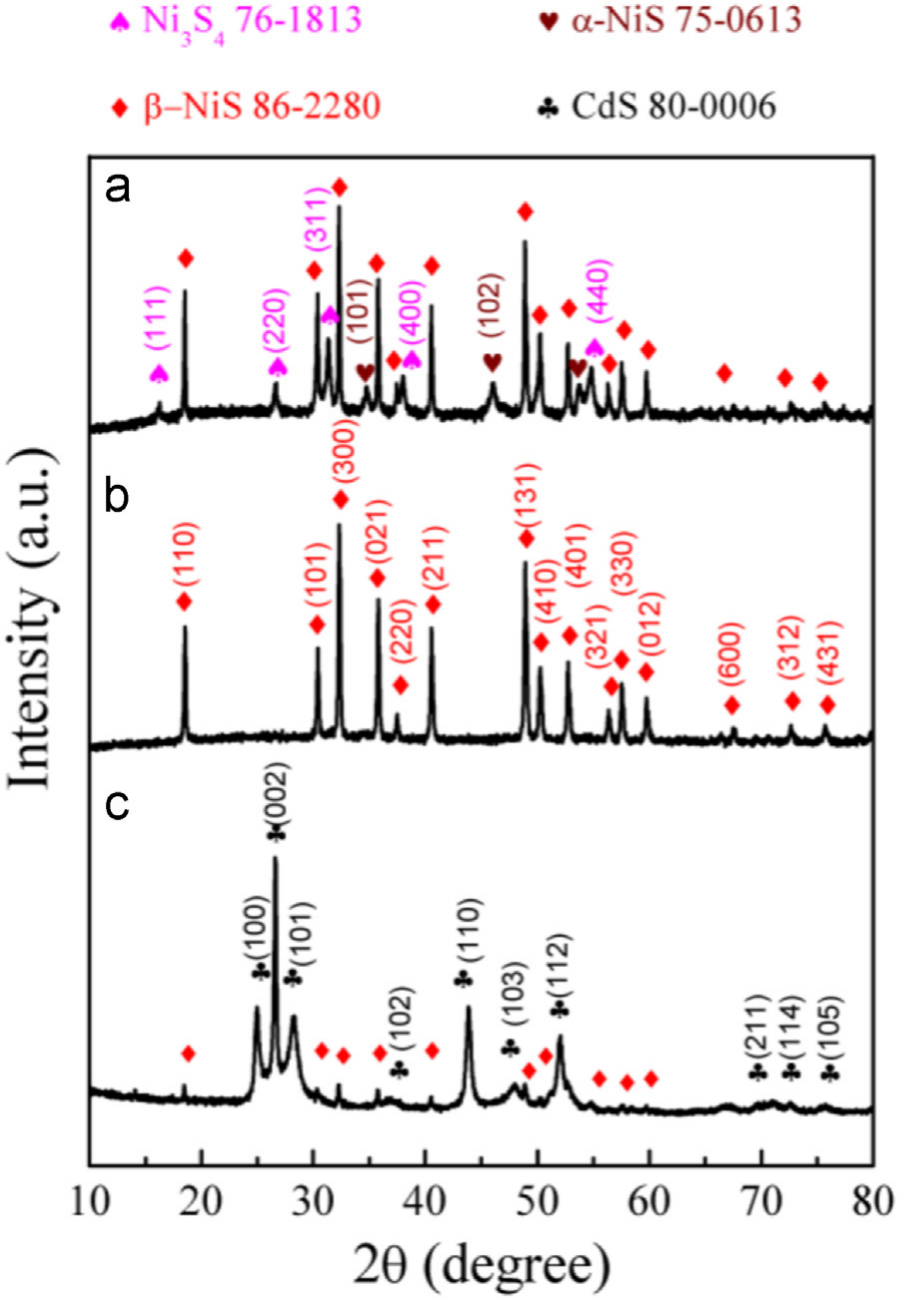
Comparison on the XRD patterns of the samples prepared under the conditions listed in Table 1d (a), Table 1e (b) and Table 1g (c).

**Fig. 10:**
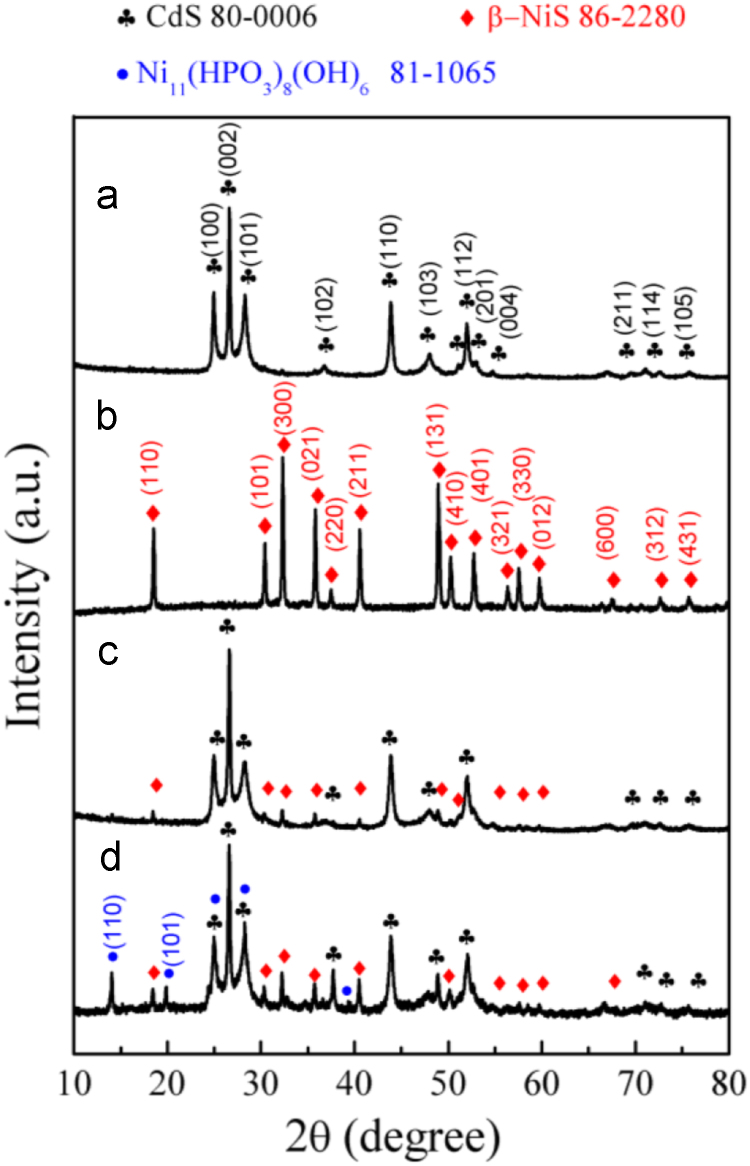
Comparison on the XRD patterns of the samples prepared under the conditions listed in Table 1a (a), Table 1e (b), Table 1g (c) and Table 1f (d).

**Fig. 11:**
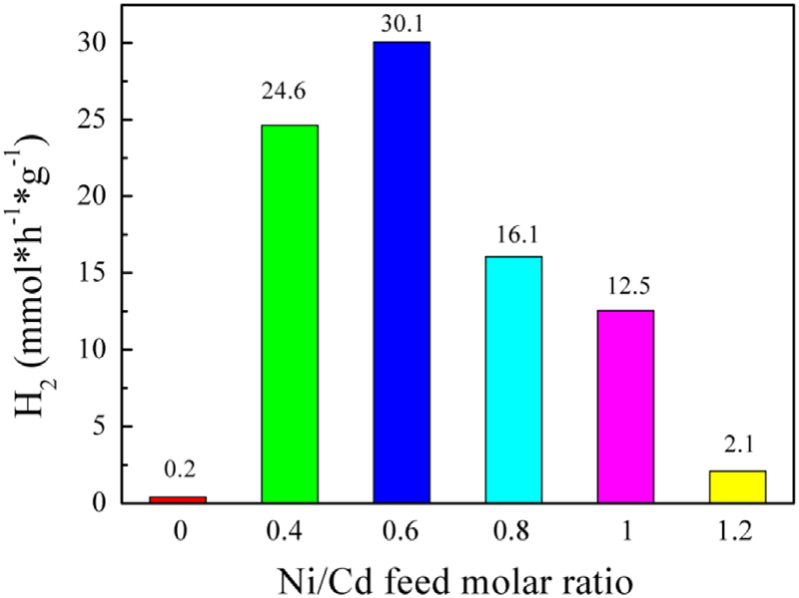
Photocatalytic activity of the NiS/CdS NFs HSNCs prepared with 0.6 mmol NaH_2_PO_2_·H_2_O and different FMRs of Ni/Cd, while the amount of Cd(NO_3_)_2_·4H_2_O was fixed at 1 mmol. For comparison, that of pure CdS NFs is also presented. Photocatalytic reaction conditions: 20 mg of the photocatalysts was dispersed in a 100 ml of aqueous solution containing 20 vol% lactic acid; the light was provided by a Xe lamp (300 W) with an UV cut-off filter (*λ* ≥ 420 nm); and the reaction cell was kept at 25 °C by cooling water.

**Fig. 12:**
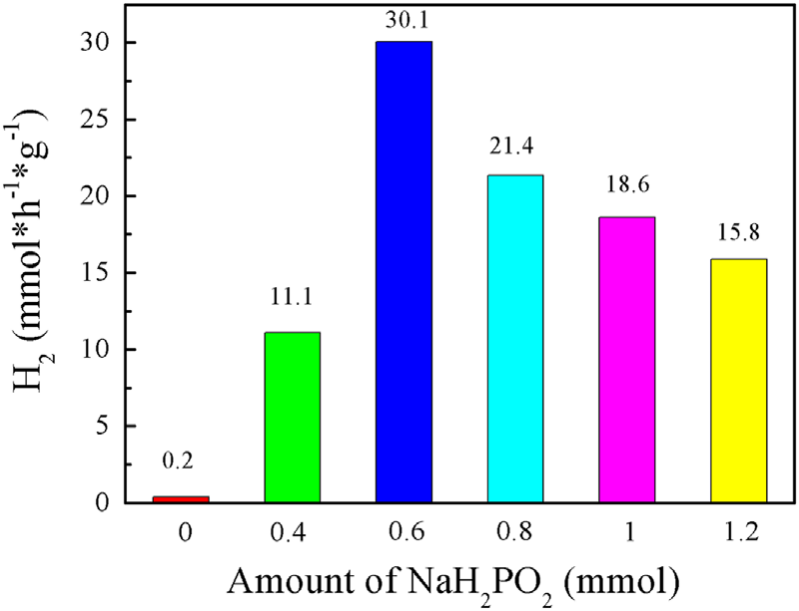
Photocatalytic activity of the NiS/CdS NFs HSNCs photocatalysts prepared with a constant Ni/Cd FMR of 0.6 and different amounts of NaH_2_PO_2_·H_2_O. Photocatalytic reaction conditions: 20 mg of photocatalyst was added into 100 ml of aqueous solution containing 20 vol% of lactic acid; a Xe lamp (300 W) with an UV cut-off filter was used to provide the light (*λ* ≥ 420 nm); and the reaction cell was kept at 25 °C with cooling water.

**Fig. 13:**
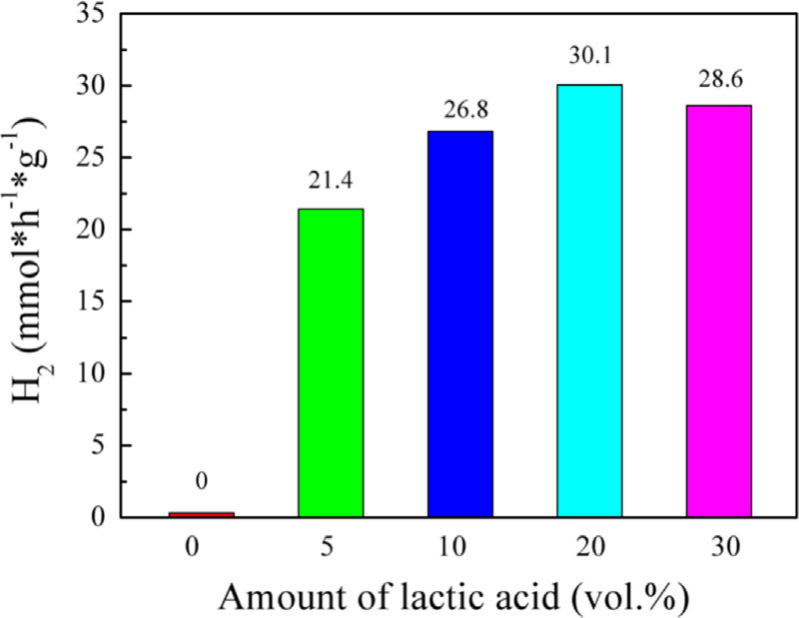
Photocatalytic activity of the NiS/CdS NFs HSNCs prepared with 0.6 mmol of NaH_2_PO_2_·H_2_O and a Ni/Cd FMR of 0.6 at different concentrations of lactic acid. Other photocatalytic reaction conditions: 20 mg of the photocatalysts was dispersed in a 100 ml of aqueous solution; the light was provided by a Xe lamp (300 W) with an UV cut-off filter; and the reaction cell was kept at 25 °C by cooling water.

**Fig. 14:**
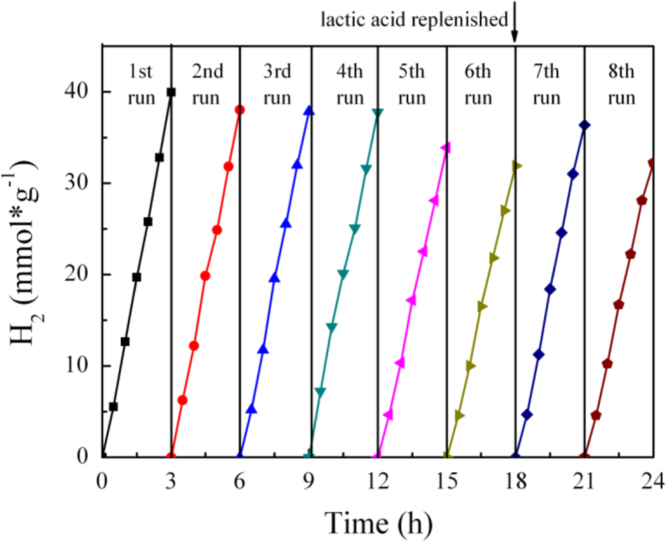
Cycling runs for photocatalytic hydrogen evolution over the optimal NiS/CdS NFs HSNC photocatalyst under light irradiation (*λ* ≥ 420 nm) provided by a 300 W Xe lamp with an UV cut-off filter condition. During the test, additionally 5 ml of lactic acid was added into the reaction cell from seventh cycle.

**Fig. 15:**
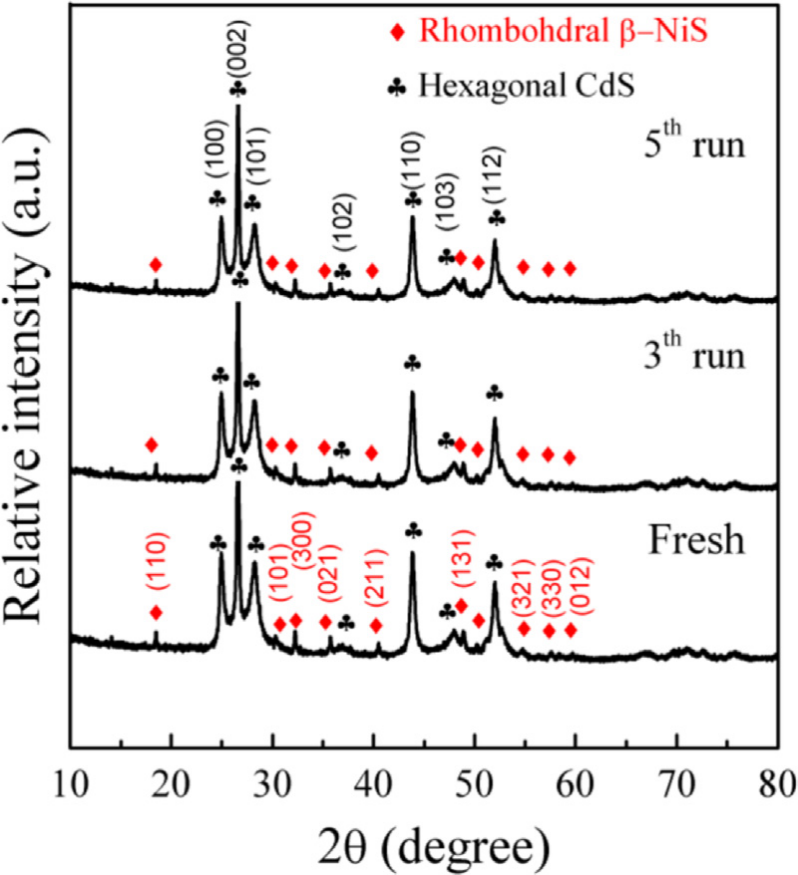
Comparison on the XRD patterns of the fresh and used NiS/CdS NFs HSNC after 9 runs of cycling test.

**Fig. 16:**
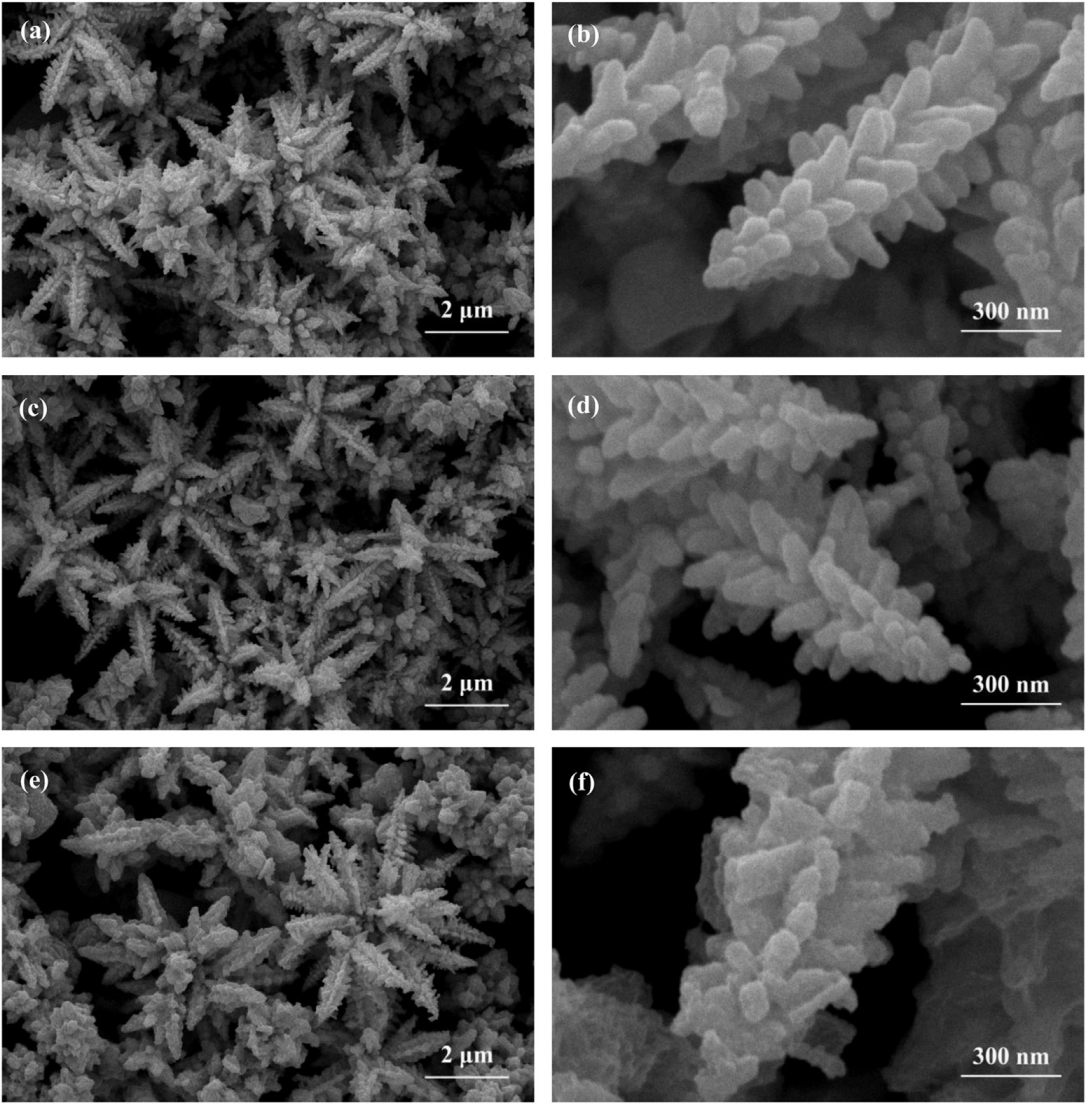
Comparison on the low-magnification and high-magnification SEM images of the fresh (a and b), and used NiS/CdS NFs HSNC after 3 (c and d) and 5 (e and f) runs of cycling tests.

It includes data on the synthesis processes optimization and formation mechanism of the present NiS/CdS NFs HSNC (Fig. 1, Fig. 2, Fig. 3, Fig. 4, Fig. 5, Fig. 6, Fig. 7, Fig. 8, Fig. 9, Fig. 10 and Table 1), which reveal that a β-NiS thin film was coated onto the CdS NFs, behaving like a film. It also includes data on the optimization on the photocatalytic activity of the NiS/CdS NFs HSNCs prepared with different Ni/Cd feed molar ratios (FMRs) from 0 to 1.2 (Fig. 11) and different amounts of NaH_2_PO_2_·H_2_O from 0 to 1.2 (Fig. 12) under different concentrations of lactic acid from 0 to 30 vol% (Fig. 13). Data on the photocatalyitc stability of the NiS/CdS NFs HSNC are also presented in Fig. 14, Fig. 15, Fig. 16. In addition, data on the activity of CdS-based photocatalysts containing inorganic cocatalysts for H_2_ production are compared in Table 2. The data set can be also used by researchers interested in developing new composite photocatalysts in other fields such as solar energy conversion, biosensing and biomedical applications, and environmental remediation [2], [3], [4], [5], [6].

**Table 1:** Dependence of the reaction resources on the composition and structure of the final products.^a^

Reaction resources (mmol)				Composition and structure of the products (sample order)	
Cd(NO_3_)_2_·4H_2_O	CH_4_N_2_S	Ni(CH_3_COO)_2_·4H_2_O	NaH_2_PO_2_·H_2_O		
1	3	0	0	Pure CdS NFs	(a)
1	3	0.6	0	Pure CdS NFs	(b)
0	3	0.6	0	No precipitate collected	(c)
0	6	1.2	0	Composite of Ni_3_S_4_ and NiS	(d)
0	3	0.6	0.6	Pure NiS NPs	(e)
1	3	1.2	0.6	Composite of NiS, CdS and Ni_11_(HPO_3_)_8_(OH)_6_	(f)
1	3	0.6	0.6	Composite of NiS and CdS	(g)

a Reaction conditions: the reaction resources were dissolved into 50 ml of distilled water, transferred into a Teflon-liner autoclave, and then reacted at 180 °C for 4 h.

**Table 2:** CdS-based photocatalysts containing inorganic cocatalysts for H_2_ production.

**Photocatalyst**	**Cocatalyst**	**Light source**^a^	**Aqueous reaction solution**	**Reaction temperature (°C)**	**H**_**2**_**evolution**			**Ref.**
					**Activity (μmol h**^**−1**^ **g**^**−1**^**)**	**QE (%)**	**Enhancement factor**^b^	
CdS nanoflowers	Β-NiS film	*λ* ≥ 420 nm (Xe)	20 vol% Lactic acid	25	30,053	43.1 (420 nm)	139	[1]
CdS polyhedral nanocrystal	NiS nanoparticles	*λ* ≥ 420 nm (Xe)	30 vol% Lactic acid	35	28,600	60.4 (420 nm)		[9]
CdS particle	NiS nanoparticles (1.2 mol%)	*λ* ≥ 420 nm (Xe)	30 vol% Lactic acid	25	7266	51.3 (420 nm)	35	[8]
CdS nanowire	NiS nanoparticles (5 mol%)	*λ* ≥ 420 nm (Xe)	0.35 M Na_2_S and 0.25 M Na_2_SO_3_		1131			[10]
sphere-like CdS	NiS nanoparticles (0.14 wt%)	250 W (Xe)	0.57 M Na_2_S and 0.4 M Na_2_SO_3_	25	5835		33	[11]
NiS/CdS nanoparticles	PbS nanoparticles (0.16 mol%)	*λ* ≥ 400 nm (Xe)	30 vol% Lactic acid		18,750		1.86	[12]
CdS nanoparticles	NiS	*λ* ≥ 420 nm (Xe); 500 W	10 vol% Lactic acid		24,370	12.78 (420 nm)	4	[13]
CdS nanorod	Fe^3+^ doped NiS_2_ nanosheet	*λ* ≥ 420 nm (Xe); 150 W	10 vol% methanol	25	3200		46	[14]
CdS pyramids	NiS nanoparticles	*λ* ≥ 420 nm (Xe)	10 vol% Lactic acid		49,200	74.6 (420 nm)		[15]

a Xe: xenon lamp.

b The enhanced factor was calculated from the activity enhancement of the photocatalysts loaded with the optimal amount of cocatalysts, in comparison with the photocatalysts without the loading of cocatalysts.

## 2 Experimental design, materials and methods

Novel high-performance NiS/CdS NFs HSNC photocatalyst was described by Zhang et al. [1]. In order to obtain the photocatalyst with strong adhesion between NiS and CdS, on the basis of improving its photocatalytic activity, the synthesis processes were optimized by adjusting the Ni/Cd FMR from 0 to 1.2 and the amount of NaH_2_PO_2_·H_2_O from 0 to 1.2 mmol under different concentrations of lactic acid from 0 to 30 vol%. All the prepared samples were characterized by XRD, SEM and TEM. And all the experiments were conducted in duplicates.

As is seen from this figure, all the XRD patterns present sharp diffraction peaks, indicating the relatively high crystallinity of all the samples. The XRD pattern of pure CdS NFs could be indexed to the hexagonal CdS phase (JCPDS card no. 80-0006). When a small amount of NiS (with a Ni/Cd FMR no larger than 0.4) was loaded onto CdS NFs, no obvious diffraction peaks belonging to NiS phase could be observed, and the patterns of such samples show no significant difference from that of CdS NFs, probably because the amount of NiS in the samples was too small, which was lower than the detection limit of XRD. However, when the Ni/Cd FMR increased up to 0.6, the diffraction peaks of NiS and CdS phases could be identified from the XRD patterns, revealing that more NiS co-existed with CdS NFs in the composites. And it was found that all the diffraction peaks of the NiS phase were ascribed to those of the rhombohedral β-NiS phase (JCPDS card no. 86-2280). With even larger Ni/Cd FMR, an impurity phase of Ni_11_(HPO_3_)_8_(OH)_6_ (JCPDS card no. 81-1065) could be identified in the samples, together with β-NiS and CdS phases, which is the result of excessive nickel resource in reaction with NaH_2_PO_2_·H_2_O. Hence, in this work, the optimal Ni/Cd FMR was chosen as 0.6 (also based their photocatalytic activities presented in Fig. 11).

As is seen from this figure, no appreciable morphology change could be observed with increasing Ni/Cd FMR for the synthesis of the samples. All the samples were mainly composed of nanoflowers. Furthermore, a lot flower-like architecture with short dendrites can be observed, which might be the inchoate state of the nanoflowers (see the white square as show in Fig. 2c, d and f).

It can be seen that the XRD pattern of NiS-CdS NFs synthesized without NaH_2_PO_2_·H_2_O (see Table 1b) is the same as that of pure CdS NFs synthesized just with Cd(NO_3_)_2_·4H_2_O and CH_4_N_2_S (Table 1a). This result indicates that the addition of NaH_2_PO_2_·H_2_O is beneficial for the synthesis of β-NiS. Furthermore, the XRD patterns of NiS/CdS NFs HSNCs with the addition of NaH_2_PO_2_·H_2_O could be indexed to the rhombohedral β-NiS and hexagonal CdS phases, but only a slight increase of the β-NiS content could be observed with further increasing addition amount of NaH_2_PO_2_·H_2_O owing to the limited, fixed amount of Ni(CH_3_COO)_2_·4H_2_O.

This figure indicates that the NiS/CdS NFs HSNCs are composed of a lot of nanoflowers, which are similar with pure CdS NFs, implying that the addition of NaH_2_PO_2_·H_2_O almost has no effect on the morphology of the NiS/CdS NFs HSNCs.

As is seen from the XRD pattern in Fig. 8a, pure hexagonal CdS phase could be directly synthesized with sufficient amount of Cd(NO_3_)_2_·4H_2_O and CH_4_N_2_S due to the strong coordinating ability of CH_4_N_2_S (see Table 1a). From the XRD pattern in Fig. 8b, it is seen that when additionally 0.6 mmol Ni(CH_3_COO)_2_·4H_2_O joined into the reaction system of Cd(NO_3_)_2_·4H_2_O and CH_4_N_2_S, a precipitate of CdS NFs almost without NiS was obtained, because the added amount of Ni(CH_3_COO)_2_·4H_2_O was too less to form NiS (see Table 1b). However, from the XRD pattern in Fig. 8c, a composite of hexagonal CdS and rhombohedral β-NiS phases can be obtained by adding 0.6 mmol NaH_2_PO_2_·H_2_O into the reaction system of Cd(NO_3_)_2_·4H_2_O, Ni(CH_3_COO)_2_·4H_2_O and CH_4_N_2_S (see Table 1g), indicating that the addition of NaH_2_PO_2_·H_2_O is beneficial for the synthesis of β-NiS.

As can be seen from Table 1c, in absence of Cd(NO_3_)_2_·4H_2_O, no precipitate can be obtained just with the same amount of 0.6 mmol Ni(CH_3_COO)_2_·4H_2_O and 3 mmol CH_4_N_2_S. However, also without applying Cd(NO_3_)_2_·4H_2_O but via doubling the concentrations of Ni(CH_3_COO)_2_·4H_2_O and CH_4_N_2_S in the reaction system (see Table 1d), a composite of cubic Ni_3_S_4_ (JCPDS card no. 76-1813), rhombohedral β-NiS and hexagonal α-NiS (JCPDS card no. 75-0613) would be obtained, as shown in the XRD pattern of Fig. 9a.

On the other hand, when a little amount of NaH_2_PO_2_·H_2_O (here 0.6 mmol) was applied in the reaction system as presented in Table 1e, pure β-NiS could be synthesized through using a small amount of Ni(CH_3_COO)_2_·4H_2_O and CH_4_N_2_S as done in the test of Table 1c (see Fig. 9b). This fact further indicates that the addition of NaH_2_PO_2_·H_2_O is beneficial for the synthesis of β-NiS phase.

Furthermore, a composite of hexagonal CdS and rhombohedral β-NiS phases can be obtained by adding 0.6 mmol NaH_2_PO_2_·H_2_O into the reaction system of Cd(NO_3_)_2_·4H_2_O, Ni(CH_3_COO)_2_·4H_2_O and CH_4_N_2_S (see Table 1g and Fig. 9c), indicating that the addition of NaH_2_PO_2_·H_2_O is beneficial for the synthesis of β-NiS, and the formation of β-NiS has no effect the synthesis of CdS phase.

Pure CdS phase (see Fig. 10a) can be obtained by the reaction system as listed in Table 1a, and pure β-NiS phase (see Fig. 10b) can be obtained by the reaction system as in Table 1e. When all the chemicals joined together into a reaction system in an appropriate ratio as the listed Table 1g, a composite (see Fig. 10c) of hexagonal CdS and rhombohedral β-NiS phases can be obtained, indicating the feasibility of the synthesis of NiS/CdS NFs HSNCs by one-step solvothermal route.

However, from the diffraction pattern shown in Fig. 10d, it can be seen that an impurity phase of Ni_11_(HPO_3_)_8_(OH)_6_ would appear when doubling the amount of Ni(CH_3_COO)_2_·4H_2_O into the reaction system to that of Table 1g, indicating that although NaH_2_PO_2_·H_2_O could promote the formation of β-NiS, it should be in an appropriate ratio with Ni(CH_3_COO)_2_·4H_2_O.

As can be seen from this figure, the hydrogen evolution rate (HER) of pure CdS NFs was rather low (0.216 mmol h^−1^ g^−1^), while over the NiS/CdS NFs HSNCs photocatalyst, the HER increased dramatically first and declined gradually later with increasing FMRs of Ni/Cd, presenting a HER of 30.1 mmol h^−1^ g^−1^ when the Ni/Cd FMR of 0.6, which is about 139 times higher than that of CdS NFs alone and much higher than those over all the CdS-NiS composite catalysts in literature (see Table 2).

The sample synthesized without NaH_2_PO_2_·H_2_O has a low photocatalytic activity similar to the pure CdS, implying that there is no NiS loading on the CdS NFs in accordance with the result of Table 1b. The HER over the NiS/CdS NFs HSNC synthesized with NaH_2_PO_2_·H_2_O first presents a sharp increase due to the enhanced loading amount of β-NiS film onto the CdS NFs, when an increasing amount of NaH_2_PO_2_·H_2_O was applied. But a gradual decrease was observed when much more NaH_2_PO_2_·H_2_O was applied, because excessive NaH_2_PO_2_·H_2_O may have a bad effect on the contact between the β-NiS film and CdS NFs owing to its reducibility on electroless plating Ni.

This figure reveals that the optimum concentration of lactic acid for the photocatalytic H_2_ evolution over the present NiS/CdS NFs HSNC photocatalyst is 20 vol%.

From this figure, it is seen that without replenishing lactic acid, the HER over the present photocatalyst under the designed light irradiation would slightly decrease continuously. Therefore, during the test, additional lactic acid was mixed into the reaction cell from 7th cycle. It was found that, after a small amount of 5 ml lactic acid was replenished in reaction system, there was an obvious increase in HER, which is similar with the report in Ref. [7]. Afterwards, the HER would restore to decrease slightly. All these results reveal that the consumption of lactic acid in the reaction system should contribute to the decrease of HER over the present catalyst, although the decomposition of the NiS/CdS NFs HSNC photocatalyst by lactic acid might also play a role [8].

This figure reveals that, compared with that of the fresh sample, there is no obvious difference for the XRD patterns of the used NiS/CdS NFs HSNC after 3 and 5 runs of cycling tests, confirming the good stability of NiS/CdS NFs HSNC photocatalyst under the designed reaction conditions.

It is seen that, after cycling test, the morphologies of the used catalyst (c-f) presented almost no change compared with that of the fresh sample (a and b), indicating that the as-synthesized catalyst is very stable under the designed reaction conditions.
