# Supplementary material for: Data on the synthesis processes optimization of novel β-NiS film modified CdS nanoflowers heterostructure nanocomposite for photocatalytic hydrogen evolution
Source: Data Brief. 2017 Dec 16;16:828–42. doi: 10.1016/j.dib.2017.12.016 (PMC5848068; doi:10.1016/j.dib.2017.12.016)
Supplement: Supplementary file 1 — Transparency document [file mmc1.pdf]

Conflicts of Interest Statement

DIB-D-17-01168

Manuscript title: Data on the synthesis process optimization of novel β-NiS film modified CdS nanoflowers heterostructure nanocomposite for photocatalytic hydrogen evolution

The authors whose names are listed immediately below certify that they have NO affiliations with or involvement in any organization or entity with any financial interest (such as honoraria; educational grants; participation in speakers' bureaus; membership, employment, consultancies, stock ownership, or other equity interest; and expert testimony or patent-licensing arrangements), or non-financial interest (such as personal or professional relationships, affiliations, knowledge or beliefs) in the subject matter or materials discussed in this manuscript.

Author names:

Yu Zhang, Zhijian Peng, Shundong Guan, Xiuli Fu

The authors whose names are listed immediately below report the following details of affiliation or involvement in an organization or entity with a financial or non-financial interest in the subject matter or materials discussed in this manuscript. Please specify the nature of the conflict on a separate sheet of paper if the space below is inadequate.

Author names:

由 扫描全能王 扫描创建

This statement is signed by all the authors to indicate agreement that the above information is true and correct (*a photocopy of this form may be used if there are more than 10 authors*):

Author's name (typed)	Author's signature	Date
Yu ZHANG	YuZhang	Nov 30, 2017
Zhijian PENG	Zhijian Peng	Nov 30, 2017
Shundong GUAN	Shundong Guan	Nov 30, 2017
Xiuli FU	Xiuli fu	Nov 30, 2017

由 扫描全能王 扫描创建
